# Antimicrobial Synergism Toward *Pseudomonas aeruginosa* by Gallium(III) and Inorganic Nitrite

**DOI:** 10.3389/fmicb.2020.02113

**Published:** 2020-08-31

**Authors:** Anna C. Zemke, Cody J. Madison, Naomi Kasturiarachi, Linda L. Pearce, James Peterson

**Affiliations:** ^1^Division of Pulmonary, Allergy and Critical Care Medicine, Department of Medicine, University of Pittsburgh, Pittsburgh, PA, United States; ^2^Environmental and Occupational Health, Graduate School of Public Health, University of Pittsburgh, Pittsburgh, PA, United States

**Keywords:** biofilm, Ga^3+^, gallium(III), nitrite, nitric oxide, *Pseudomonas aeruginosa*

## Abstract

The ubiquitous involvement of key iron-containing metalloenzymes in metabolism is reflected in the dependence of virtually all bacteria on iron for growth and, thereby, potentially provides multiple biomolecular targets for antimicrobial killing. We hypothesized that nitrosative stress, which induces damage to iron metalloproteins, would sensitize bacteria to the ferric iron mimic gallium(III) (Ga^3+^), potentially providing a novel therapeutic combination. Using both laboratory and clinical isolates of *Pseudomonas aeruginosa*, we herein demonstrate that Ga^3+^ and sodium nitrite synergistically inhibit bacterial growth under both aerobic and anaerobic conditions. Nitric oxide also potentiated the antimicrobial effect of Ga^3+^. Because many chronic pulmonary infections are found as biofilms and biofilms have very high antibiotic tolerance, we then tested the combination against biofilms grown on plastic surfaces, as well as the apical surface of airway epithelial cells. Ga^3+^ and sodium nitrite had synergistic antimicrobial activity against both biofilms grown on plastic and on airway epithelial cell. Both Ga^3+^ and various NO donors are (independently) in clinical development as potential antimicrobials, however, we now propose the combination to have some particular advantages, while anticipating it should ultimately prove similarly safe for translation to treatment of human disease.

## Introduction

Antibiotic resistance, especially in relation to the management of nosocomial infections, continues to be a growing problem worldwide. This matter is of some particular concern in the treatment of chronic suppurative lung diseases such as cystic fibrosis (CF). *Pseudomonas aeruginosa* is the most common Gram-negative pathogen in adults with CF, and decades of antibiotic exposure leads to acquired antibiotic resistance in addition to the high innate antibiotic resistance of the organism (Patient Registry, 2011). Beyond its prominence in CF, *P. aeruginosa* is one of the *ESKAPE* pathogens, causing a variety of respiratory infections and chronic wound infections, including those at surgical sites, that can be recalcitrant to treatment (Rice, 2010; Mulani et al., 2019). Thus, there is clearly an ongoing need for the development of new antimicrobial approaches toward *P. aeruginosa*.

Iron is a required nutrient for growth of nearly all bacteria, certainly including pathogens like *P. aeruginosa*. Moreover, iron is a main growth limiting nutrient in sputum (Goss et al., 2018). There is intense competition for iron in the airway, with the bacterium producing siderophores to scavenge iron and the host attempting to sequester available iron (reviewed in Palmer and Skaar, 2016). Once imported by the bacterium, iron becomes incorporated into a wide variety of metalloenzymes, including those of the bioenergetic pathways and core carbon metabolism such as NADH dehydrogenase (complex I), aconitase and various other dehydrogenases, dehydratases and reductases. Because of the critical requirement of iron for *P. aeruginosa* growth, blocking iron uptake or metabolism has been discussed as a possible treatment approach (Banin et al., 2006). The Ga^3+^ and Fe^3+^ ions have similar radii and other chemical properties, allowing Ga^3+^ to be an iron mimetic in biologic systems (Chitambar, 2016). Ga^3+^ is imported via a subset of *P. aeruginosa* iron uptake systems, and incorporated into metalloproteins in the place of iron (García-Contreras et al., 2013). As Ga^3+^ is unable to be reduced to Ga^2+^ under physiologic conditions, Ga^+3^ incorporation leads to catalytically inactive holoproteins. The full physiologic effects of Ga^3+^ on bacterial metabolism are still being determined, however, it is known that in *P. aeruginosa*, Ga^3+^ is bound by the siderophore pyoverdine and, as the Ga^3+^ cannot be reduced, it traps pyoverdine preventing its recycling (Kaneko et al., 2007; Yeterian et al., 2010). Additionally, growth in Ga^3+^ leads to decreased catalase activity (or potentially decreased expression), with a consequent increase in susceptibility to oxidative stress and decrease in ribonucleotide reductase activity (Goss et al., 2018). Ga^3+^ is currently in drug development for treatment of *P. aeruginosa* infections in CF (nebulized Ga^3+^ citrate, Aridis Pharmaceuticals). The recently published IGNITE study, which used a molar equivalent solution of gallium nitrate and sodium citrate dihydrate, provides proof of concept for the therapeutic approach (Goss et al., 2018).

One approach being studied to increase the biologic activity of Ga^3+^ is by complexing Ga^3+^ with protoporphyrin and starving the bacterium of iron with the addition of the iron chelator deferiprone (Richter et al., 2017). Alternatively, increasing turnover of iron metalloproteins might increase susceptibility to Ga^3+^. Nitrosative stress, specifically peroxynitrite derived from nitric oxide, causes damage to Fe-S clusters. Nitric oxide and acidified (pH 6.5) nitrite, which is metabolized to nitric oxide, are both being tested as antimicrobial approaches for chronic airway infections in CF (clinicaltrials.gov locators NCT02694393 and Howlin et al., 2017). Note that pH 6.5 is being used in our studies to mimic airway mucus conditions in CF (Yoon et al., 2006), but NO is readily generated from the nitrite anion at pH 7.4 *in vivo* (Cambal et al., 2011, 2013).

Given the common broad target of iron biochemistry, we hypothesized that sodium nitrite and Ga^3+^ salts may have synergistic antimicrobial activity. Nitric oxide, produced from nitrite, should cause widespread damage to iron-containing proteins resulting in, for example, increased turnover of iron-sulfur (Fe-S) proteins. If Ga^3+^ were available, it would be incorporated in the place of Fe^3+^ during this state of increased turnover, leading to dysfunctional metalloproteins and consequent widespread bacterial metabolic arrest. Thus, nitrite-derived nitric oxide should potentiate the antibacterial effects of Ga^3+^.

## Materials and Methods

### Reagents

Reagents were purchased from Sigma Aldrich except for PAPA-NONOate (Caymen Chemicals) and Bacto Agar (BD). Solutions were made fresh immediately prior to the experiment and were not used if precipitation was present. The pH of media was confirmed to be 6.5 under all conditions tested.

### Strains and Growth Conditions

Strains were cultured in Lysogeny Broth (LB) overnight on a roller drum at 37°C prior to experimentation. The laboratory strains PAO1 and PA14 were used (obtained from George O’Toole, Dartmouth). The *P. aeruginosa* clinical isolate panel was described in Zemke et al. (2014). The PA14-*hitA:*IS strain was obtained from the PA14 non-redundant transposon library (Liberati et al., 2006). Clinical Isolates were obtained from the Cystic Fibrosis Isolate Core at Seattle Children’s Hospital.

### Checkerboard Synergy Testing

Studies were done using M-9 minimal media with glucose as a carbon source and 4μM FeCl_3_. For aerobic studies, checkerboard agar dilution plates were made with 0–1,600 μg/ml Ga(NO_3_)_3_ and 0–30 mM NaNO_2_. Overnight cultures were diluted to 5 × 10^5^ CFU/ml and spotted on plates. Plates were grown for 48 h at 37°C and scored for growth. In some cases, liquid MIC assays were done with a similar protocol using 96-well plates and optical density as the endpoint, with positive growth scored as an optical density above the sterility well. Anaerobic studies were done using M9-glucose plates with 1% KNO_3_ to support anaerobic respiration. For anaerobic studies, strains PAO1 and PA14 were diluted, spotted on plates, and the plates were incubated in GasPak jars for 4 days prior to scoring. Plates were scanned at high resolution on an HP Scanner in groups of 8 and images were scored based on if growth was visible on the image. Images were stored so that they could be reviewed by a second scorer if needed. The Fractional Inhibitory Concentration (FIC) was used to define synergy. FIC was calculated with the following formula: FIC = (MIC_Acombo_/MIC_A_ + MIC_Bcombo_/MIC_B_), where A represents Ga(NO_3_)_3_ and B represents NaNO_2_. Synergy was defined as FIC <0.5; no interaction is defined as FIC > 0.5 to = 4 (Petersen et al., 2006).

### Abiotic Biofilm Prevention Assay

Abiotic biofilms were grown on plastic microtiter plates as described in Zhang and Mah (2008). Overnight cultures were rinsed with M-9 media twice prior to dilution to remove residual LB broth. Biofilms were grown in pH 6.5 M-9 media with glucose as a carbon source and 4μM FeCl_3_ for 24 h, then stained with crystal violet and visually examined for growth. Ga(NO_3_)_3_ and NaNO_2_ were added at the beginning of the experiment. Data were analyzed by two methods. First, the wells were photographed and scored for visible growth. In the second method, the crystal violet was dissolved in acetic acid and the optical density was determined at 570 nm. The background was subtracted and wells were scored based on an 80–90% reduction in OD as compared to the control well. For a well to be considered the MIC, next two adjacent wells were required to be scored negative for growth. Seven total replicates were done.

### Biotic Biofilm Assays

The biotic biofilm dispersal protocol was modified from Moreau-Marquis et al. (2008). The strain PAO1 was used for these experiments. The human airway epithelial cell line CFBE41o- was grown at air-liquid interface on Transwell filters. Epithelial cells were grown submerged for 48 h after seeding, and then grown at air-liquid interface for an additional 5–12 days. Cells were fed through the basolateral compartment. The day of the experiment, filters were rinsed three times on both apical and basolateral compartments to remove any residual antibiotics present from the growth medium. Detailed descriptions of this assay are found in Moreau-Marquis et al. (2010). Filters were inoculated at a Multiplicity of Infection of 25:1. After 6 h of growth, the apical and basolateral compartments were rinsed twice with phosphate buffered saline (PBS), pH 6.5 to remove residual free amino acids present in the cell culture media. Biotic biofilms were treated with 50 mM NaNO_2_ and Ga(NO_3_)_3_ for 60 min. Bacteria were counted by serial dilution, and the limit of detection was 100 CFU/ml. Five replicates were done. We confirmed epithelial barrier function integrity through the measurement of trans-epithelial electrical resistance with an EVOM device as described in Zemke et al. (2014). Filters were allowed to equilibrate with PBS on both sides for 60 min, then nitrite and/or gallium(III) was applied to the apical surface and the resistence was measured after 60 min. At least three replicates were done for each condition.

### Statistical Analysis

At least three replicates were done of all experiments. Statistical analysis was done using PRISM 8.0 software (GraphPad, San Diego California). Data are displayed as mean ± standard deviation. CFU counts were log transformed, and then one-way ANOVA was used.

## Results

### Nitrite and Ga^3+^ Have Synergistic Antimicrobial Activity

**FIGURE 1:**
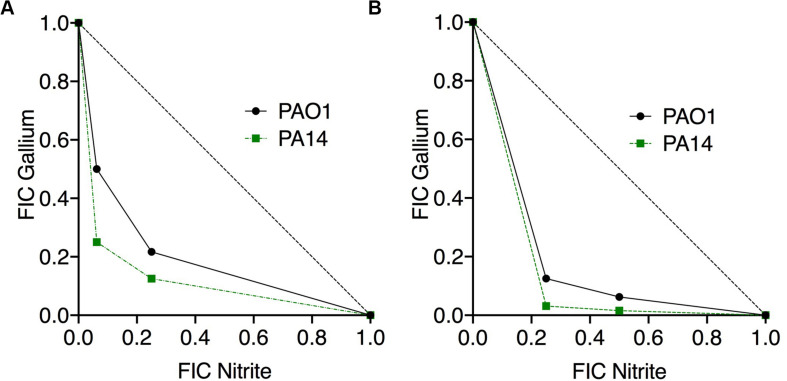
Nitrite and Ga^3+^ have synergistic antibacterial activity. Isobolograms show the results of checkerboard assays using PAO1 and PA14 presented showing the fractional inhibitory concentration (FICs) of the two compounds in combination under aerobic **(A)** and anaerobic **(B)** conditions. Six aerobic replicates done, three anaerobic replicates done, representative isobologram shown.

If nitrite and Ga^3+^ are targeting iron metalloprotein-dependent metabolism through complementary mechanisms, we would predict that the two compounds would display antimicrobial synergy. Therefore, we performed checkerboard testing to determine the Fractional Inhibitor Concentration (FIC) of Ga^3+^ and nitrite for both laboratory strains and CF clinical isolates of *P. aeruginosa*. Minimum Inhibitory Concentrations (MICs) and FIC values for the laboratory strains PAO1 and PA14 grown aerobically on glucose are shown in Table 1. The concavity of isobolograms showing the relationship between the nitrite and Ga^3+^ FICs for PAO1 and PA14 are visually indicative of synergy (Figure 1A), and the Ga^3+^-nitrite FIC for both strains was < 0.5, meeting the definition of antimicrobial synergy (Petersen et al., 2006). We determined the Ga^3+^-nitrite FICs for a panel of CF *P. aeruginosa* isolates. The Ga^3+^ MICs ranged from 6 to 96 μM, while the MIC for nitrite was 15 mM for all isolates (Table 1). In the isolate panel, 11/16 strains displayed synergy. Anaerobic growth, such as that found in the CF lung, causes increased antimicrobial tolerance as well as reliance on alternative metabolic pathways which may have different sensitives to inhibition by Ga^3+^ and nitrite (Alvarez-Ortega and Harwood, 2007; Arai, 2011; Schaible et al., 2012). Under anaerobic conditions, the Ga^3+^ MIC increased to 100–200 μM for PAO1 and 800–1,600 μM for PA14. The anaerobic nitrite MIC was 5 mM for both strains and, again, synergy was seen for both strains (Figure 1B). While the MICs for Ga^3+^ and nitrite varied with oxygen availability, the compounds were clearly synergistic under both aerobic and anaerobic conditions.

**TABLE 1:** Checkerboard assay for nitrite-Ga(III) interaction for *P. aeruginosa* grown aerobically on M9 media with glucose as a carbon source.

Strain	Ga MIC (μM)	NIT MIC (mM)	Ga MIC (μM) (3.25 mM NIT)	FIC
PAO1	12	15	3	0.47
PA14	24	15	6	0.47
Strain 31-1	12	15	3	0.47
Strain 31-2	24	15	24	1.22
Strain 33-2	24	15	12	0.72
Strain 36-2	24	15	12	0.72
Strain 36-3	12	15	3	0.47
Strain 41-2	24	15	6	0.47
Strain 47-2	24	15	6	0.47
Strain 47-3	96	15	24	0.47
Strain 60-2	48	15	12	0.47
Strain 60-3	24	15	6	0.47
Strain 66-1	24	15	24	1.22
Strain 66-2	48	15	12	0.47
Strain 71-1	24	15	6	0.47
Strain 71-2	24	15	6	0.47
Strain 74-1	12	15	6	0.72
Strain 74-2	6	15	1.5	0.47

### Nitric Oxide Is the Agent Responsible for the Observed Synergism With Ga^3+^

**FIGURE 2:**
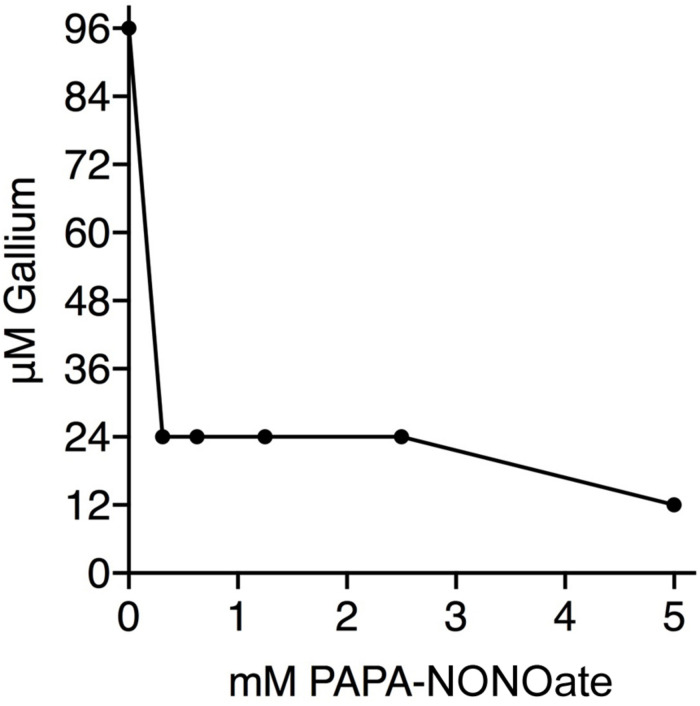
Nitric Oxide and Ga^3+^ have synergistic antibacterial activity. Isobologram shows the results of aerobic checkerboard assays using PA14. The MIC for PAPA-NONOate was >5 mM. Three replicates done.

The nitrite anion is reduced to NO within minutes upon administration (ip or iv) to mammals (Cambal et al., 2011, 2013). *P. aeruginosa* also reduces nitrite to nitric oxide, including in CF airway surface liquid (Yoon et al., 2006). Consequently, our working hypothesis is that NO is the active antimicrobial agent. Therefore, it was important to show that similar results were achievable with an alternative NO donor to nitrite. Addition of PAPA-NONOate to 312 μM dropped the MIC for Ga^+3^ from 96 μM to 24 μM (Figure 2) demonstrating the observed synergy to be qualitatively independent of the particular NO donor species. The half-life of PAPA-NON-oate is 15 min at 37°C min and that of nitrite *in vivo* is just a few min (Cambal et al., 2011, 2013). Thus, a comparatively brief exposure to NO is responsible for potentiating the antimicrobial activity of Ga^3+^ against *P. aeruginosa*. The synergistic consequences, however, are evident during the subsequent 24 hr of bacterial growth in the assays.

### Nitrite and Ga^3+^ Have Anti-biofilm Activity

**FIGURE 3:**
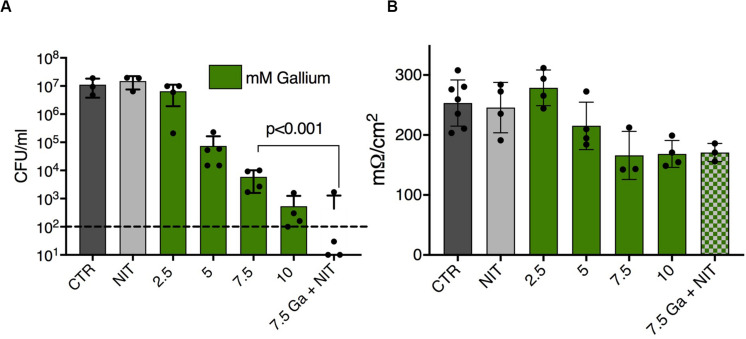
Ga^3+^ and nitrite have synergistic antibiofilm activity. **(A)** Biofilm growth prevention assays done with PAO1 grown in PVC microtiter disks and stained with crystal violet. Asterisks indicated well scored with no growth. Four replicates done. **(B)** Representative results of a biofilm prevention assay in which final optical density of the crystal violet was measured spectrophotometrically. Highlighted cells show growth as defined as <20% reduction in OD compared to the control well. Three replicates were done.

**FIGURE 4:**
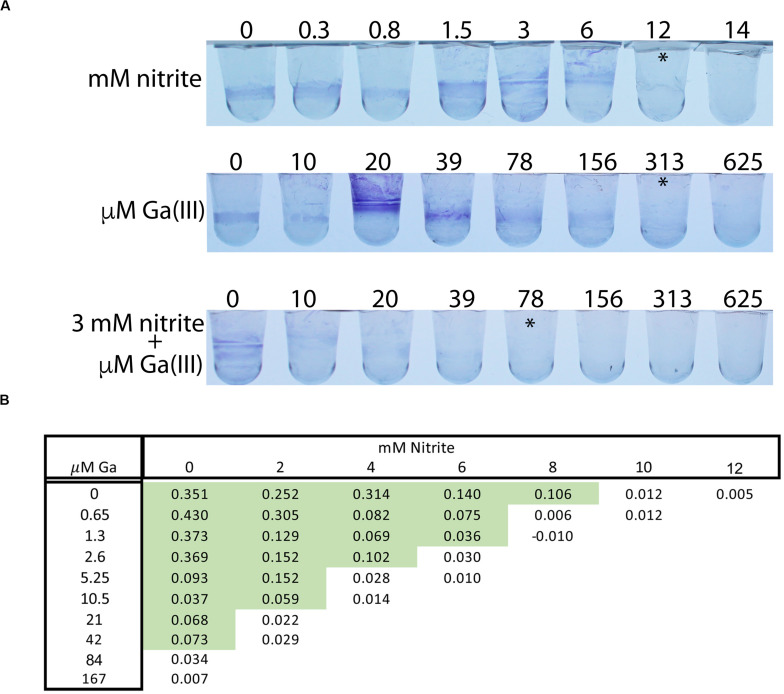
**(A)** PAO1 biofilms grown on CF airway epithelial cells were treated for 90 min with Ga or NIT for 90 min and live CFUs were plated. Ga^3+^ shows dose dependent bacterial killing that is increased with the addition of nitrite. Dashed line is assay detection limit. **(B)** Tran-epithelial electrical resistance of airway cells treated with the indicated combinations for 60 min. *p* < 0.001 by one-way ANOVA followed by *post hoc* test. Replicates done: 3–4/condition tested. ^∗^Indicates visual MIC value.

Growth conditions have wide ranging effects on bacterial physiology, and biofilms specifically have high antimicrobial tolerance in many settings, including growth in the human airway. We therefore tested the interaction between nitrite and Ga^3+^ against *P. aeruginosa* biofilms grown on polyvinyl chloride. In this assay, 14 mM nitrite prevented PAO1 biofilm growth, as did 313 μM Ga^3+^ (asterisks, Figure 3A). In the presence of 3 mM nitrite, 37 μM Ga^3+^ prevented biofilm growth, giving an FIC < 0.5. Using OD_570_ as a quantitative endpoint, we determined the concentration required to decrease biofilm formation by at least 80% (Figure 3B). A representative checkerboard result is shown, where the MIC for nitrite was 10 mM, the MIC for gallium(III) was 84 μM, which dropped to 5.25 μM in the presence of 4 mM nitrite. Biofilm growth in the presence of airway epithelial cells can be associated with even higher antimicrobial tolerance, thus we tested the combination in a model where *P. aeruginosa* biofilms are grown on the apical surface of the human airway epithelial cell line CFBE41o-(Moreau-Marquis et al., 2009). Biofilms were grown for 6 h and then treated for 90 min with 75 mM sodium nitrite or Ga^3+^. We observed a dose dependent reduction in CFU with increasing concentrations of Ga^3+^. No decrease in CFU was seen with nitrite, consistent with prior observations that nitrite is bacteriostatic under these conditions. The addition of nitrite to 7.5 mM Ga^3+^ reduced CFU to below the limit of detection. Trans-epithelial electrical resistance of the did not drop until 7.5 mM Ga^+3^ and the addition of nitrite did not potentiate the drop in resistance (Figure 4B). In summary, we saw additional antibiofilm activity with addition of nitrite to Ga^3+^ in biofilms grown on plastic, as well as those grown on airway epithelial cells.

### Nitrite Also Sensitizes Otherwise Ga^3+^ Resistant Isolates

**FIGURE 5:**
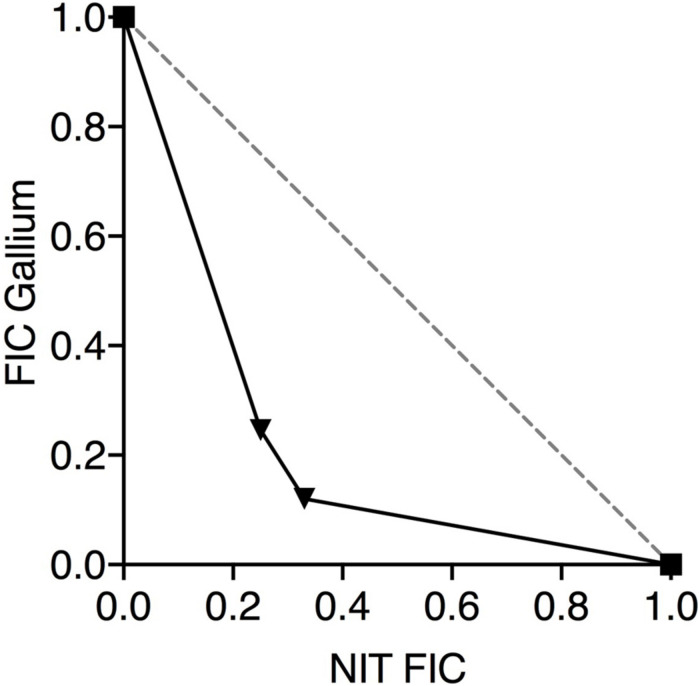
Decreased Ga intake raises MIC but synergy is preserved. Isobolograms show the results of checkerboard assays using PA14 *hitA:*IS under aerobic conditions. Three replicates done.

The main route for bacterial uptake of Ga^3+^ is the iron transporter HitAB, and two independent studies have demonstrated that loss of HitAB function is the predominant route for development of Ga^3+^ resistance (García-Contreras et al., 2013; Goss et al., 2018). Insertional disruption of *hitA* increased the Ga^3+^ MIC to 150 μM (compared with 24–48μM for the parental strain). The nitrite MIC was 12 mM for PA14 *hitA:*IS, and the addition of 3 mM nitrite dropped the Ga^3+^ MIC to 37μM (Figure 5). These results are consistent with those of the more Ga^3+^ resistant clinical isolates, where nitrite also lowered the Ga^3+^ MIC (Table 1).

## Discussion

Ga^3+^, nitric oxide and sodium nitrite are all independently in early development as antimicrobial approaches for cystic fibrosis. NO is a gas with an inconveniently short half-life making delivery problematic, although approaches such as nocturnal delivery and NO release polymers are being investigated (Reighard and Schoenfisch, 2015; Howlin et al., 2017). Sodium nitrite has the potential advantage that it can be dosed less frequently via nebulization (Simon et al., 2016). While there has not been convincing efficacy data from these compounds to date, the existing safety data is reassuring (Miller et al., 2012; Simon et al., 2016). Ga^3+^ has broad antimicrobial activity, including growth inhibition of *P. aeruginosa, Rhodococcus equii* (Harrington et al., 2006), *Mycobacterium tuberculosis* (Olakanmi et al., 2000) *Acinetobacter baumannii* (de Léséleuc et al., 2014), and *Staphylococcus aureus* (Baldoni et al., 2010). Subcutaneous administration of Ga^3+^ maltolate rescued thermally injured mice from infection with *P. aeruginosa* (DeLeon et al., 2009). Proof-of-concept studies in patients were completed using a well-tolerated intravenous formulation of Ga^3+^ as an antimicrobial in cystic fibrosis (Goss et al., 2018). No decrease in sputum bacterial density was seen, however, lung function improved with the treatment. An inhaled formulation of Ga^3+^citrate is currently being tested in individuals with cystic fibrosis (ClinicalTrials.gov identifier NCT03669614). Given the existing sets of safety data, further translational studies are feasible.

Bacterial replication is essential for most bacterial pathogenesis, and core metabolic pathways such as oxidative phosphorylation, DNA synthesis, denitrification and the Krebs cycle are rich in iron-metalloproteins, particularly those containing Fe-S clusters. These cofactors seem to be more stable in mammalian enzymes than their bacterial counterparts, and consequently, agents causing widespread Fe-S cluster damage may prove useful antimicrobials (Pearce et al., 2005, 2009). Both nitrite and Ga^3+^ have been proposed separately as antimicrobial agents targeting core bacterial metabolism, but the idea of using them together in a combination therapy seems to be a new development. Almost certainly, peroxynitrite generated secondary to the NO-donor activity of nitrite will be the agent primarily responsible for any widespread damage to iron-containing proteins. It is anticipated that, in many instances, the more rapid reproduction of the pathogen compared to host cells will further ensure that the bacterial metabolism is more significantly affected by the treatment than that of the host. Consistent with this proposition, we have shown that Ga^3+^ and nitrite-derived nitrosative stress have synergistic antimicrobial activity against *P. aeruginosa* under aerobic and anaerobic conditions (Figures 1, 2). More encouragingly still, nitrite and Ga^3+^ are also synergistic in preventing biofilm growth on plastic (Figure 4), and the addition of nitrite to Ga^3+^ increases the disruption of biofilms grown on human airway epithelial cells (Figure 3).

The specific bacterial protein targets of combined nitrite and Ga^3+^ exposure are presently not identified but are almost certainly multiple and will likely vary between species (Peterson et al., in preparation). Media carbon source influences *P. aeruginosa* susceptibility to Ga^3+^, probably due to reliance on metabolic pathways differing with carbon source (Rzhepishevska et al., 2011). The net effect of nitrite plus Ga^3+^ under varying conditions will likely reflect a combination of individual enzymes susceptibilities to nitrosative damage, their comparative efficiencies of regeneration and the essentiality of any single enzyme under the particular conditions. Additionally, our results do not exclude a model where nitrosative stress creates a state of iron deprivation exacerbated by Ga^3+^ quenching the principal siderophore pyoverdine (Kaneko et al., 2007; Yeterian et al., 2010). Resistance to Ga^3+^ occurs through loss of *hitAB*, which is the principal uptake transporter, as well as through increased expression of pyocyanin (García-Contreras et al., 2013). While inactivation of *hitAB* led to Ga^3+^ resistance, the MIC for nitrite was unchanged, and nitrite lowered the MIC to Ga^3+^ comparably to the parental strain; suggesting that this multi-targeting, synergistic approach may render the development of resistance less problematic. The effects of Ga + 3 and nitrite on the development of resistance remain to be experimentally determined.

In summary, we have demonstrated that inorganic nitrite and Ga^3+^ have synergistic antibacterial activity against *P. aeruginosa* under a range of test conditions and in biofilms. These findings appear to be novel in that they use a double attack on multiple targets within core bacterial metabolism with inexpensive and stable compounds that could feasibly be moved into human testing. The choices of particular nitrite compound(s) to be employed and the specific gallium(III) salt(s) serving as a source of Ga^3+^ could be dependent upon pharmacodynamic and pharmacokinetic considerations not addressed here. The compounds will also require *in vivo* toxicity testing in combination. The most direct application of these finding might be the combination of systemic gallium, which is already FDA approved, with topical or inhaled nitrite formations that are capable of safely achieving high local concentrations of nitrite. Another attractive alternative is the combination of inhaled nitrite or gaseous NO with gallium maltolate, which can be administered orally. Ultimately, though, new syntheses should not be required, since there are probably enough pre-existing compounds available for combining into suitable formulations.

## Data Availability Statement

The raw data supporting the conclusions of this article will be made available by the authors, without undue reservation, to any qualified researcher.

## Author Contributions

AZ conducted the experiments, wrote the first draft of manuscript, and analyzed the data. CM, NK, and LP conducted the experiments. JP conceived the concept, supervised experimental conduct and data analysis, and revised the manuscript. All authors contributed to the article and approved the submitted version.

## Conflict of Interest

The authors declare that the research was conducted in the absence of any commercial or financial relationships that could be construed as a potential conflict of interest.
